# Evolvability Is Inevitable: Increasing Evolvability without the Pressure to Adapt

**DOI:** 10.1371/journal.pone.0062186

**Published:** 2013-04-24

**Authors:** Joel Lehman, Kenneth O. Stanley

**Affiliations:** 1 Department of Computer Science, The University of Texas at Austin, Austin, Texas, United States of America; 2 Department of Electrical Engineering and Computer Science, University of Central Florida, Orlando, Florida, United States of America; Chang Gung University, Taiwan

## Abstract

Why evolvability appears to have increased over evolutionary time is an important unresolved biological question. Unlike most candidate explanations, this paper proposes that increasing evolvability can result without any pressure to adapt. The insight is that if evolvability is heritable, then an unbiased drifting process across genotypes can still create a distribution of phenotypes biased towards evolvability, because evolvable organisms diffuse more quickly through the space of possible phenotypes. Furthermore, because phenotypic divergence often correlates with founding niches, niche founders may on average be more evolvable, which through population growth provides a genotypic bias towards evolvability. Interestingly, the combination of these two mechanisms can lead to increasing evolvability without any pressure to out-compete other organisms, as demonstrated through experiments with a series of simulated models. Thus rather than from pressure to adapt, evolvability may inevitably result from any drift through genotypic space combined with evolution's passive tendency to accumulate niches.

## Introduction

An unbroken hereditary chain links the simplest early replicators to the most complex modern macroscopic organisms. Observing this evolutionary trajectory raises the question of the *cause* for the appearance of increasing evolutionary potential, i.e. increasing *evolvability* [1]. Although the cause of such increase is still debated, most candidate explanations for evolvability rely on selection pressure [1]–[8], reflecting natural selection's significant explanatory power in other contexts. For example, selection on mutation or recombination rates [2], species-level selection to adapt [1], [3], selection for stability of evolved structures [3], [8], and persisting through fluctuating selective environments [5] have all been proposed as partial explanations for increasing evolvability. However, adaptive explanations may be unnecessary or at least merit more scrutiny if increasing evolvability is demonstrated *without* any pressure to adapt, that is, if evolvability results from a more fundamental (and potentially passive) process.

This paper investigates two such alternative hypotheses for evolvability. The first hypothesis is that if evolvability itself is heritable, then even a passive drifting process over genotypes will differentiate the evolvability of organisms, and the more evolvable of these organisms will be more likely to become phenotypically diverse and spread through niches. That is, a biased distribution of phenotypes can result from a passive drift over genotypes.

Intuitively, in a passive drift some mutations may increase an organism's evolutionary potential, while others may decrease it. Importantly, note that such passive drifting does not cause an inherent drive towards increasing evolvability when averaged over all genotypes in the entire population. However, it turns out that evolvability averaged *over niches* may still rise even in a purely drifting model (i.e. a model with a fixed-size population that evolves solely through genetic drift). This conclusion follows from one widely-held conception of evolvability as the capacity of an organism to “generate heritable phenotypic variation” [3], which is also the definition adopted in this paper. While evolvability is also sometimes discussed in relation to adaptation [9], [10], the chosen definition reflects a growing consensus in biology that phenotypic variability in its own right deserves study in the context of evolvability [3], [4], [6], [11]. Thus, following this definition, those organisms that are least evolvable will on average change less phenotypically from repeated mutation, while those that are more evolvable will change more, i.e. more evolvable organisms will have a higher average *velocity* of phenotypic change.

As a result, the phenotype space itself can act as a filter, whereby more evolvable organisms will be separated from the less evolvable over time as they radiate at different velocities throughout the phenotypic space. This sorting mechanism is similar to how a centrifuge or a western blot separates particles of different densities or charges. In other words, at any point in time the least evolvable organisms are most likely to be found clustered together within the phenotypic space, occupying niches near their evolutionary origins. In contrast, the more evolvable organisms are more likely to diverge phenotypically over time to inhabit niches divergent from their ancestors. Thus, even if the genotypic space is evolving without direction, the resulting distribution in the phenotypic space can still become biased towards the more evolvable. That is, uniformly sampling the genotype space (which is unbiased) would on average choose less evolvable organisms than would uniformly sampling the phenotype space (which is biased). The bias in the distribution of phenotypes is that less evolvable organisms are likely to be found densely concentrated in only a few niches (near their evolutionary origin), while the more evolvable organisms are more likely to spread *throughout* reachable niches.

Thus if a population is drifting through a genotypic space, from surveying only the phenotypic space it might be mistakenly inferred that the average evolvability over all organisms had *increased*, i.e. that there is a genotypic bias towards evolvability. Furthermore, the cause of this apparent increase might be misattributed to selection pressure. In reality, however, there is no selection pressure, and the average evolvability of genotypes will not have significantly changed: Only the average evolvability per niche (i.e. averaged over divergent phenotypes) will have increased. The interesting implication is that the deceptive appearance of increasing evolvability can result from a random walk over genotypes. However, the main insight is that evolvability may be self-reinforcing: A drifting process in the genotypic space may warp the phenotypic distribution in proportion to evolvability, and given a sufficiently large population, the *maximum* evolvability may also increase over time, which further warps the phenotypic space. Supporting this hypothesis, experiments with both an abstract mathematical model and simulated evolved machines reveal the appearance of increasing evolvability through only a drifting process.

However, while genetic drift biases only the phenotypic distribution of organisms towards greater evolvability, an additional non-adaptive mechanism may also similarly bias the *genotypic* distribution. This genotypic bias can result from the correlation between phenotypic divergence and establishing new niches. In other words, evolvable organisms may be more likely to lead to new ways of life [3]. Thus more evolvable organisms may become over-represented as founders of new niches, causing the resulting population growth from niche foundation to bias the genetic space also towards increasing evolvability. Thus the second hypothesis for non-adaptive evolvability increase is that founder effects in new niches tend to amplify more evolvable organisms on average. The end result is that *overall* evolvability, i.e. not just its appearance, may also increase over time in nature – but not due to adaptive pressure to out-compete other organisms, which is a foundational assumption that underlies many other theories for the rise of evolvability [1]–[8].

Supporting this second hypothesis, further experiments with growing populations in which evolution is initiated within a single niche, and where each niche has a limited capacity (but where selection is random within a niche) demonstrate a significant trend towards increasing *genotypic* evolvability over time. Importantly, the drive towards overall increasing genotypic evolvability in these experiments is qualitatively more substantial than in the drifting models alone (where the appearance of increasing evolvability results only when averaged over niches). Another abstract model and two additional models with evolved machines exhibit the same trend towards increasing genotypic evolvability without selection pressure for adaptation. The surprising conclusion is that increasing evolvability may not result from selective pressure to adapt, but may instead be an inevitable byproduct of how evolvability warps the distribution of phenotypes and the tendency for founding new niches to amplify evolvable organisms.

## Experiments

The next sections describe experimental models that investigate the hypotheses in this paper.

### Appearance of Increasing Evolvability in Passive Drift Models

The first set of experiments illustrate that evolvability can appear to increase as a result of a passive drifting process over genotypes. That is, if evolvability is heritable then a drifting process in a genotype space can separate the more evolvable organisms from the less evolvable ones over time, inducing a distorted distribution in phenotype space that yields the deceptive impression of overall increasing evolvability. This first hypothesis is explored in two models, a highly-abstract model and a model based on simulated evolved robots.

#### Abstract passive drift model

The highly-abstract model consists of a population of abstract organisms that evolve solely due to genetic drift (i.e. there is no selection pressure nor population growth). The idea is to investigate whether genetic drift can yield the appearance of increasing evolvability in a minimal model. Thus each organism in this model has only two hereditary properties: the niche that it occupies and its evolvability, both of which are subject to mutation. An organism's niche is represented as a two-dimensional point within a discrete grid, which mutation perturbs by shifting the point one unit in either dimension. In other words, the genotype-to-phenotype map is trivial in this model: The niche specified in an organism's genotype maps directly into its phenotypic niche (which is the two-dimensional point in the discrete grid). The evolvability of an organism is thus specified as the probability that an organism's niche will be perturbed through mutation, which reflects the assumption that more evolvable organisms have greater phenotypic variability [3], [4], [6], [11]. In contrast to the initial probability for an organism's niche to be perturbed (i.e. the organism's *initial* evolvability), evolvability itself mutates more infrequently through small perturbations (exact parameter settings can be found in the Methods Section). In other words, the assumption is that evolvability tends to evolve at slower rates than typical hereditary properties. Note that all organisms are initially identical, i.e. they begin in the same niche (in the center of the grid) with the same level of evolvability.

The idea is that as the population drifts in this model, the evolvability of each organism undergoes a random walk. Thus over time the evolvability of organisms will become differentiated as by chance mutation some become more evolvable and some become less so. However, across the entire population, evolvability will remain constant on average because increasing and decreasing are equiprobable. Concurrent with changes in evolvability, the niches of organisms are also evolving stochastically. Recall that in this model the probability of an organism's niche mutating is linked to its evolvability.

The interesting effect of such linkage is that it causes the niche space to act as a filter that over time separates the less evolvable organisms from the more evolvable. In other words, if all organisms are initialized to start from the same niche, on average the organisms that become most evolvable (by chance mutation) will also evolve to be farthest in the niche space from the starting location (because evolvability here correlates with an increased future chance of changing niches). That is, the most evolvable organisms have a higher phenotypic velocity of change. As a result, the least evolvable organisms will on average cluster near the initial niche, and the most evolvable organisms are more likely to be found along the peripheral niches. Thus by observing the distribution of evolvability across the *niche space* (which is equivalent to the phenotypic space in this simplified model) one might falsely conclude that evolvability in general has increased. In other words, the average evolvability *per niche* will have increased.

**Figure 1:**
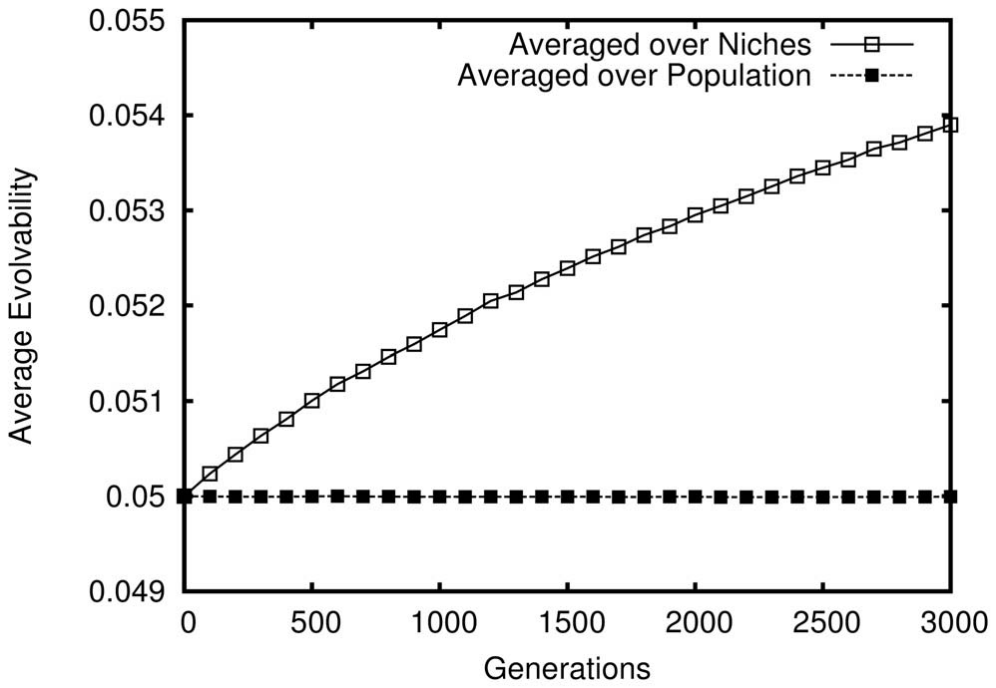
Evolvability in the abstract passive drift model. How the evolvability of organisms changes over generations is shown averaged (in different ways) over 50 independent simulations that last 3,000 generations each. If evolvability is averaged *within* each niche and then *over* all niches, then evolvability *appears* to increase. However, if instead evolvability is simply averaged over all organisms in the population, there is no significant overall increase in evolvability over time.

**Figure 2:**
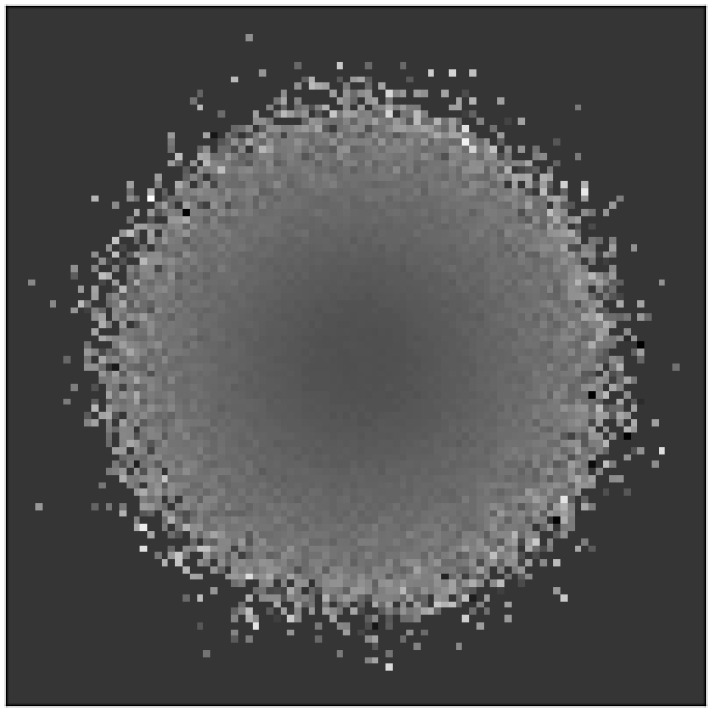
Evolvability heat map for the abstract passive drift model. The average evolvability of organisms in each niche at the end of a simulation is shown averaged over 50 independent runs. The lighter the color, the more evolvable individuals are within that niche. The overall result is that evolvability increases with increasing distance from the starting niche in the center.

**Figure 3:**
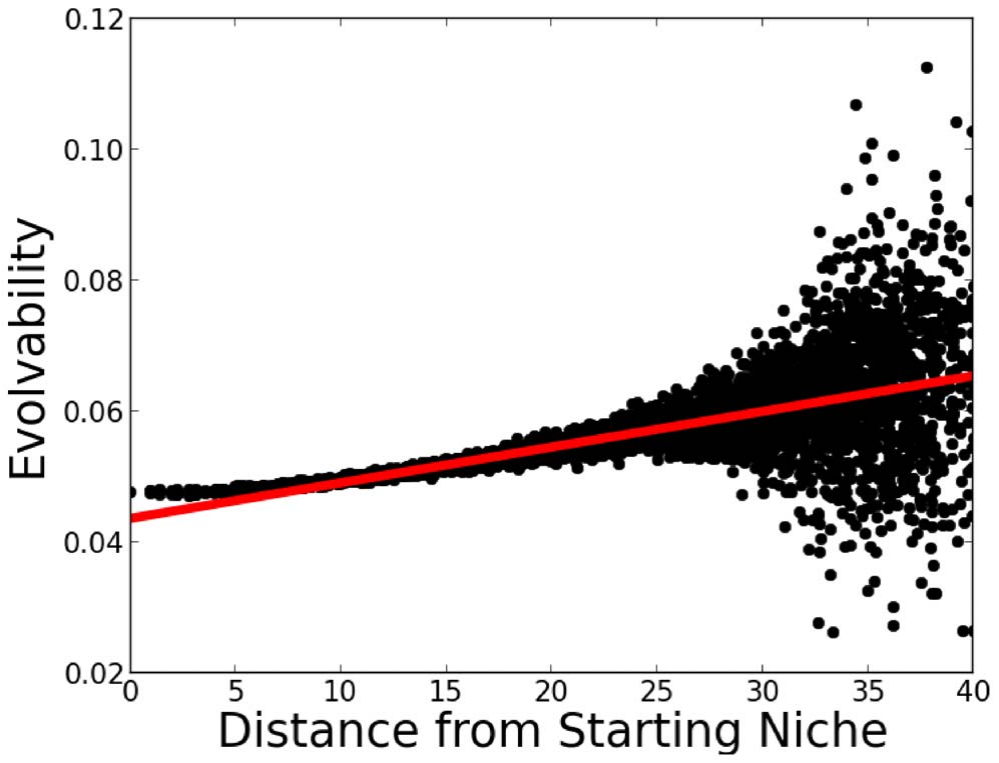
Evolvability vs. distance from the initial niche for the abstract passive drift model. The evolvability of organisms at the end of a simulation is shown as a function of distance from the initial starting niche (averaged over 50 independent runs). The main result is that there is a significant correlation between increasing distance from the initial niche and increasing evolvability in this model. The plotted line indicates the line of best fit by linear regression.

However, evolvability across the *population* remains unchanged on average; it is only evolvability's distribution over the space of *niches* that becomes biased during evolution. This bias, and the unbiased population-wide average of evolvability are shown in figure 1. More clearly illustrating the bias over the niche space, figure 2 shows a heat-map of evolvability over the grid of niches at the end of a simulation and figure 3 shows how evolvability varies as a function of a niche's distance from the starting niche. Note that there is a strong monotonic relationship between the distance of an organism from the starting niche at the end of a simulation and its evolvability (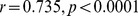; Pearson's r).

#### Passive DRIFT with evolved robots

To augment the evidence provided by the purely-abstract model in the previous section, a more concrete genetic space is considered in this model, which has a richer genotype-to-phenotype map. The idea is to begin exploring whether the results from the abstract passive drift model reflect a general tendency or if they are overly specific to parameters or assumptions in the simple model. As an initial such exploration, a genotype-phenotype mapping and simulated environment are implemented in the spirit of digital evolution [12] and evolutionary robotics (ER; [13]). In particular, a genotype-phenotype map for a simulated robot controlled by evolved artificial neural networks (ANNs) is adapted from prior ER experiments [14]–[16].

In this model, genotypes encode ANNs that control simple wheeled robots embedded in a simulated maze. The motivation is to abstract at a high level how evolved neural structures influence an organism's behavior in its environment. In other words, a genotype in this model ultimately maps to the behavior of a robot in a simulated maze environment. Though other domains could be applied, this environment is well-studied [14]–[16] and offers a non-trivial genotype-phenotype mapping. Niches in this model are specified by creating a discrete grid over a space of possible robot *behaviors*, i.e. what the robot actually does in the simulation, as opposed to a characterization of its genotype or of the ANN controller itself to which the genotype maps. So in this model, as in nature, the niche space is a many-to-one mapping from the space of phenotypes, i.e. many similar phenotypic behaviors will map into the same niche. The idea is that different classes of behaviors are what facilitate niches, not simple differentiation of genotypes or encoded neural structures. Thus a robot is mapped into a niche as a function of its behavior.

While there there is no overall consensus on how to quantify evolvability [5], a growing body of work supports that evolvability is related to phenotypic variability [3], [4], [6], [11]. Thus the evolvability of genotypes in this model (also following precedent in ER [17]), is given by quantifying the amount of behavioral variety (i.e. the number of different behavioral niches) reachable on average by random mutations from a particular genotype. In other words, an evolvable organism is more likely to lead to phenotypic divergence. Note that in this model evolvability is not directly encoded into the genotype as in the abstract model, but is a quantifiable emergent product of the genotype-phenotype map, more closely resembling the situation in biological evolution. Specific parameters and details about the evolvability measure are given in the Methods Section.

The general motivation for this model is that passively drifting with a relatively large population (e.g. on the order of millions) composed of this kind of more concrete genotype may exhibit the same appearance of increasing evolvability as observed in the abstract passive drift model. A larger population is necessary in this experiment because most random genotypes in this more realistic genotypic space represent similarly trivial behaviors, i.e. most randomly-connected ANNs encode no meaningful information, and the appearance of increasing evolvability will only emerge when drifting over a sufficient quantity of differentiated non-trivial behaviors. Thus to facilitate these experiments in a computationally efficient way, a limited genetic space of ANNs that was tractable to exhaustively characterize and explore was enumerated (i.e. a discrete subset was considered from a much larger space of ANNs with continuous weights and variable network topologies). Then each one of the enumerated genotypes in the space (which consisted of 38.7 million different genotypes) were evaluated in the maze environment to quantify its behavior and evolvability. For each simulation the population was uniformly initialized to a randomly chosen genotype (i.e. the population is always initially homogeneous) and subject to differentiating genetic drift for 250 generations. Importantly, supporting the assumption that evolvability is heritable, despite overall wide variance in evolvability over the entire genetic space, the evolvability of a parent and its offspring are well correlated (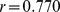; Pearson's r). The details of the particular neural model applied can also be found in the Methods Section.

**Figure 4:**
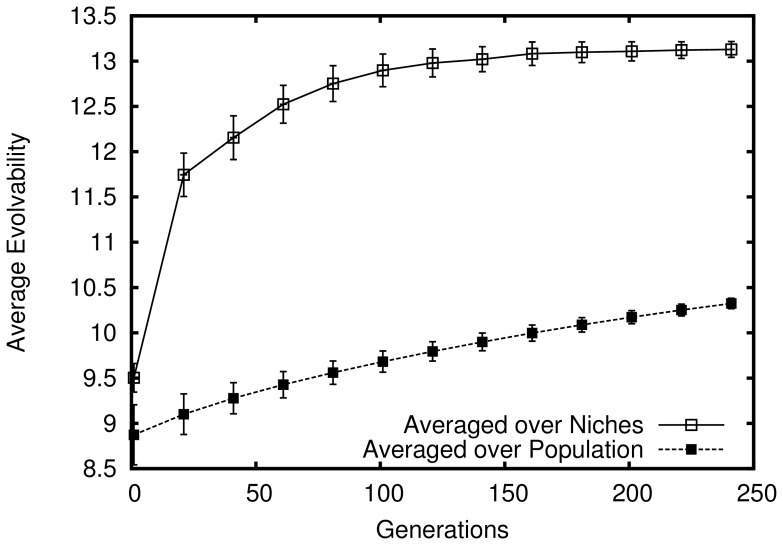
Evolvability of evolved robots with the passive drift model. The evolvability of evolved robots subject to passive drift is shown averaged (in different ways) over 50 independent runs that lasted 250 generations each. If evolvability is averaged within each niche and then over all niches, it appears to increase. If instead evolvability is averaged over all organisms, there is no significant increase.

Interestingly, a plot of evolvability over time from this model also demonstrates the appearance of increasing evolvability over time (figure 4), providing evidence that the hypothesis that genetic drift can lead to the appearance of increasing evolvability may hold true not only in abstract theoretical circumstances.

### Population Growth with Limited Capacity Niches Increases Evolvability

The previous models demonstrate how a purely random drifting process can create the deceptive appearance of increasing overall evolvability. The next experiments explore the hypothesis that a qualitatively more pervasive increase in evolvability (i.e. an overall bias towards genotypes with higher evolvability) can result from population growth and niches with limited capacity. First, an extension of the abstract passive drift model is considered.

#### Abstract model with limited capacity niches

This section considers a variation of the abstract model introduced earlier, but where the size of the population varies dynamically (previously this size was fixed). In particular, the population grows geometrically (i.e. each organism is replaced by two offspring in the next generation), but the overall size of the population remains tractable because in this model niches are limited in capacity (i.e. when a new generation is created, niches can grow only to a certain size, after which further individuals entering that niche are discarded). The idea is to roughly model the concept of limited resources in natural evolution and explore its effect on evolvability. Importantly, this extended model still imposes no *direct* selection pressure for evolvability, and if the model started at equilibrium (i.e. with all niches at full capacity), there would be no expectation of evolvability increasing over time. In other words, what is important for evolvability in this model is spreading through the space of niches. Furthermore, selection *within* a niche is purely random–there is no selection for adaptation to the niche nor any way for one organism to reliably *out-compete* another. Thus this is a model without adaptive pressure.

**Figure 5:**
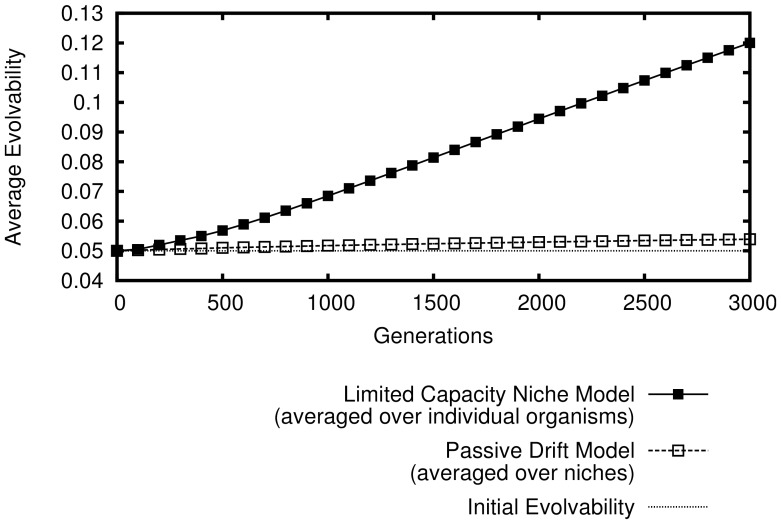
True increasing evolvability in the abstract model with limited capacity niches. How the average evolvability of organisms in the population changes over time is shown (averaged over 50 independent simulations that lasted 1000 generations each). Note that the line shown for the passive model (reproduced from figure 0) represents only the *appearance* of increasing evolvability in that model when evolvability is averaged over niches.

However, despite this lack of adaptive pressure, as evolution progresses in this model the passive filtering effect of the phenotypic space demonstrated in the fixed-sized population model is amplified. The explanation is that the resulting population growth from founding a new niche (by mutating out of the zone of previously explored niches) *indirectly* rewards increasing evolvability in this model: The more evolvable organisms (which because of their higher velocity of phenotypic change are more likely to mutate into new niches) are continually amplified from population growth as they diffuse through niches. Thus as more niches are discovered and filled, the population becomes increasingly biased towards evolvability; in effect, the reward for discovering a new niche in this model accelerates the filtering process that is purely passive in the passive drifting model. This acceleration is shown in figure 5, which compares evolvability in this model to that of the passive drift model introduced earlier. Note that the figure shows growth in overall evolvability (i.e. averaged over all organisms) in the limited capacity niche model that greatly outpaces the growth in the passive drift model (which is only significant when averaged over niches). In other words, superficial niche-level evolvability in the passive drift model grows more slowly than evolvability over all genotypes in the limited capacity niche model. This result is important because it demonstrates a true increase in average evolvability over a population without selection pressure to out-compete other organisms.

**Figure 6:**
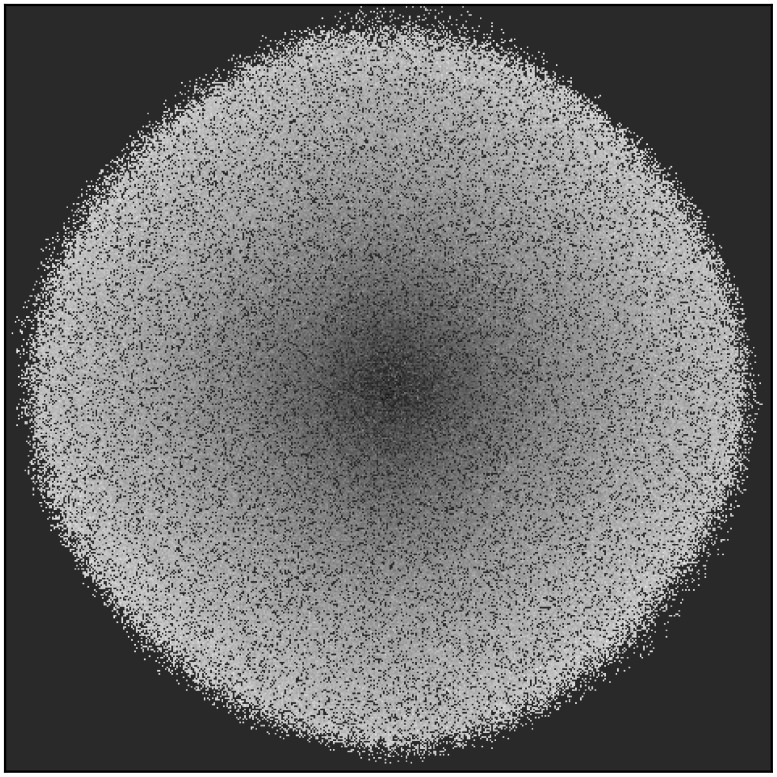
Evolvability heat map for the abstract model with limited capacity niches. The average evolvability of organisms in each niche at the end of a simulation is shown. The lighter the color, the more evolvable individuals are within that niche. The overall result is that, as in the first model, evolvability increases with increasing distance from the starting niche in the center.

**Figure 7:**
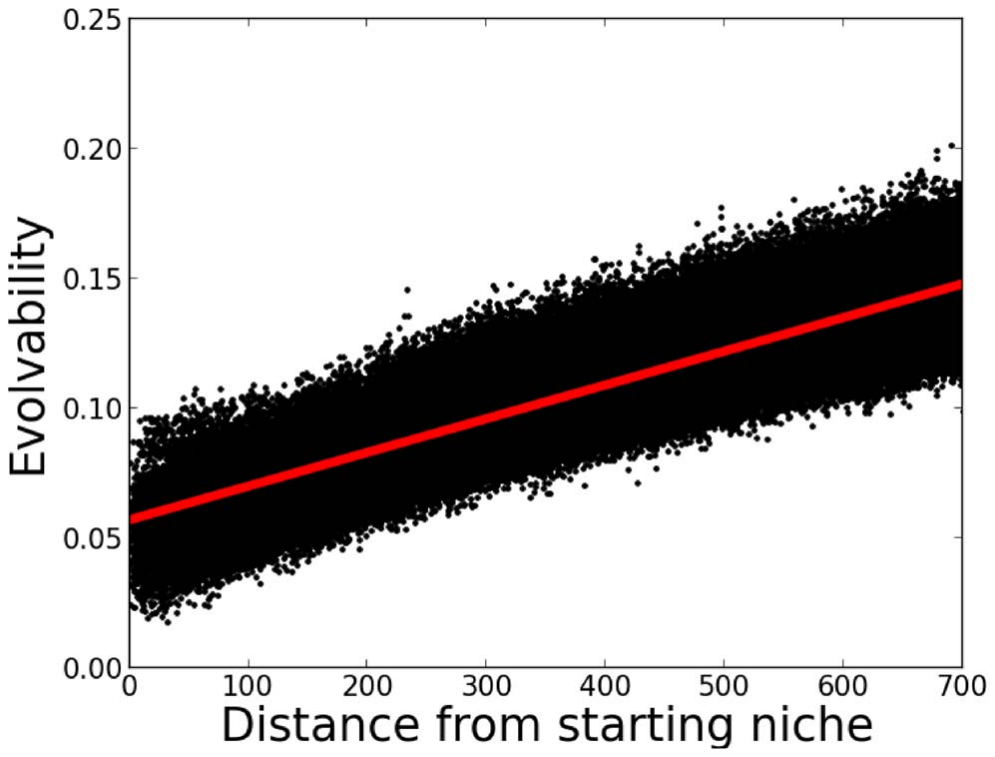
Evolvability vs. distance from the initial niche for the abstract model with limited-capacity niches. The average evolvability of organisms in the final population is shown as a function of distance from the initial starting niche averaged over 50 independent simulations. The main result is that there is a significant correlation between increasing distance from the initial niche and increasing evolvability. The plotted line indicates the line of best fit by linear regression.

Similarly to the first model, figure 6 shows a heat-map of evolvability over the niche space averaged over all runs, and figure 7 shows how evolvability varies over the niche space as a function of a niche's distance from the starting niche. There is a strong significant monotonic relationship between the distance from starting niche and evolvability (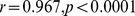; Pearson's r).

#### Evolved robots model with limited capacity niches

**Figure 8:**
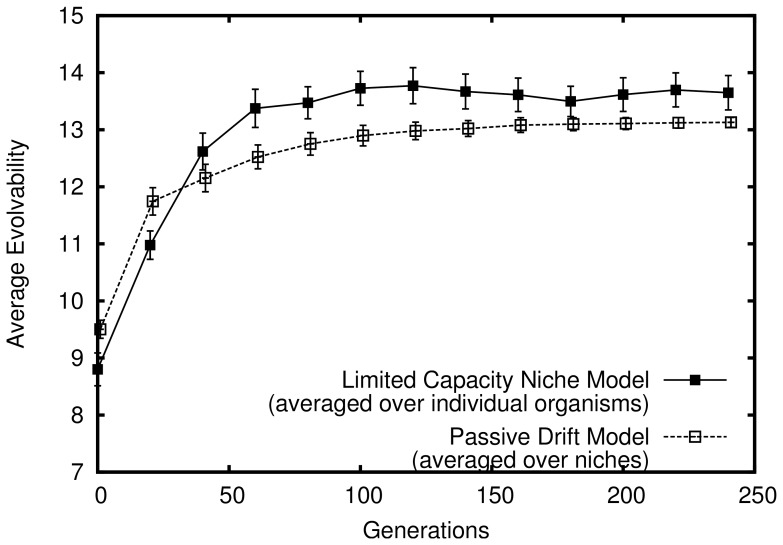
True increasing evolvability in the evolved robots model with limited capacity niches. The evolvability of organisms is shown averaged over 50 independent simulations that lasted 250 generations each. It is important to note that because the population size in the limited-niche model is much smaller, *one* generation of the passive model encompasses more individuals (2,000,000) than is considered over *all* generations in the niched model (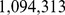 on average). In other words, the limited-niche model is more directedly and more efficiently biased towards evolvability. In particular, the difference in evolvability between the limited-niche model and the passive model is significant for all comparisons after the 50th generation (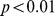; Student's t-test).

Like the previous extension to the abstract model, this section extends the drifting model with simulated evolved robots to include population growth and limited capacity niches. The idea is to explore whether this more realistic genotype-phenotype mapping will also exhibit the same *accelerated* increase of evolvability seen from extending the abstract model. The results of this experiment are shown in figure 8, and confirm that limiting niche capacity also biases population growth more strongly towards increasing evolvability in this more concrete genetic space. In other words, the limited niche capacity models (both the abstract model and the model with evolved robots) demonstrate a bias towards true evolvability (i.e. when averaged over the entire population) while the passive drift models exhibit a weaker bias towards evolvability that is significant only when averaged over niches.

#### Practical ER model with limited capacity niches

Finally, the last model explores evolvability in a less restricted space of ANNs. The idea is to examine whether the hypotheses in this paper hold even in a commonly used practical ER system. In particular, this model explores a limited-capacity niched model (as in the previous two experiments) but with a well-established practical neuroevolution method called NEAT [18]. Instead of having only three discrete settings for ANN connection weights (i.e. inhibitory, excitatory, or neutral), the connection weights in NEAT can vary continuously in strength. Additionally, to facilitate increasingly complex evolved behaviors, the topology of the ANN can itself become increasingly complex because of mutations that incrementally introduce new connections and nodes during evolution. As a result of continuous weights and increasing complexity, the space of ANNs that NEAT explores is effectively infinite and cannot be fully enumerated as in the previous model. However, the benefit is that this class of genetic space is more analogous to that provided by DNA in nature, which is also open-ended in a similar way.

Because the space cannot be fully enumerated due to computational limits, it is impossible to fully characterize the genotypic space and precisely calculate evolvability (as was done in the fully passive model). This full characterization of the space also facilitated efficient simulation of millions of evolved robots through a precomputed look-up table that mapped a genotype to its niche and evolvability. Such a table is impossible to construct for the practical ER model, and thus only the limited niche capacity setup is implemented here (because it exhibits a driven trend towards evolvability increase that is not dependent on a large population size). As in the prior robot controller models, the niche space consists of a discretized grid of the possible locations to which a robot can navigate within the maze. The evolvability of a genotype is estimated (because the space cannot be exhaustively enumerated) by counting how many phenotypic niches are reachable from many independent random mutations of the original genotype. As a control to show that niching robots in a structured way is having a positive effect on evolvability, a comparison experiment is also run where niching is random (i.e. an ANN's niche is specified by a random number generator instead of being derived from the robot's behavior). In other words, the random control does not consistently reward behaviors that are different from those already present in the system.

**Figure 9:**
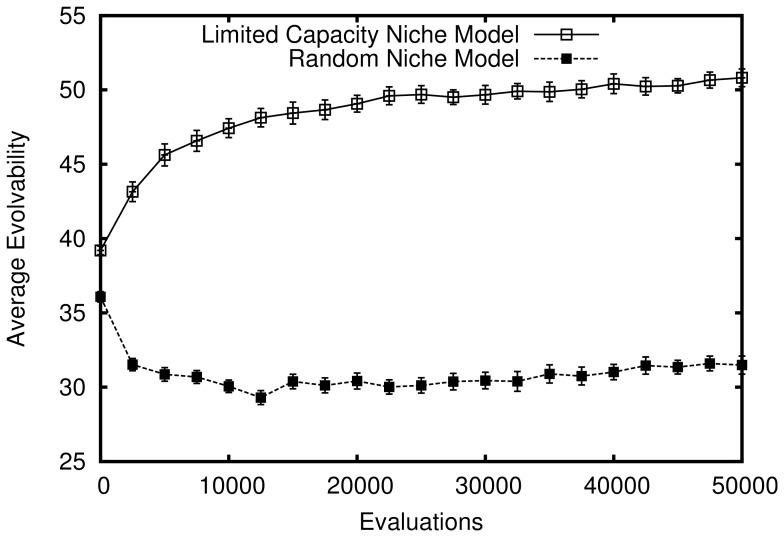
Evolvability in the practical ER model with limited capacity niches. The average evolvability of organisms in the population over evolutionary time is shown, which is itself averaged over 50 independent simulations that lasted 50,000 evaluations each. Note that evolvability of an organism is measured as the average number of different behaviors generated through 300 random mutations. The main result is that niching based on behavior significantly increases evolvability over a control that randomly assigns niches independently of an organism's behavior (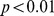 for all comparisons after 2,500 evaluations; Student's t-test).

The results of these experiments are shown in figure 9, which reinforce the hypotheses in this paper by similarly demonstrating the benefits for evolvability of population growth with limited niche capacity in a more realistic genetic setting. Note that as in the previous two experiments, limiting niche capacity encourages true evolvability growth.

## Conclusions

This paper presented evidence for two non-adaptive explanations for the appearance of increasing evolvability over the course of biological evolution. The first is that an unbiased drifting process over genotypes can nonetheless produce a distribution of phenotypes (where multiple individuals in the population may have the same phenotype) biased towards increasing evolvability, and the second is that founder effects for discovering niches can provide a genotypic bias towards true evolvability increase. While such non-adaptive explanations do not contradict more popular adaptive explanations, they call them into question because the mechanisms shown here require fewer assumptions, i.e. they result from the structure of the genotype-phenotype map and founder effects from uncovering new niches instead of particular transient selective pressures. In fact, the results from the passive drift models suggest even caution in the assumption that evolvability in general has truly increased over evolutionary time; the superficial *appearance* of overall increasing evolvability can result from evolvable organisms filling a larger volume of phenotypic space.

However, even assuming that evolvability has truly increased, these results still illustrate the danger in habitually viewing evolution through the lens of selection pressure. An alternative perspective through which to interpret the results is to view evolution as a process driven to diversity as it expands through new niches. Such niche expansion is a ratcheting process, whereby niches rarely go unfilled after being discovered. The founder effect and population growth from uncovering new niches serve to bias the genotypic space towards increasing evolvability because they amplify genomes that diverge phenotypically, which on average tend to be those that are more evolvable. Thus if the assumption of evolvability's heritability holds, then such founder effects in establishing new niches may yield a persistent bias towards increasing evolvability – even in the absence of adaptive competition between organisms.

In this view increasing evolvability may simply be an inevitable result of open-ended exploration of a rich genetic space. Importantly, in nature this passive drive towards evolvability may have bootstrapped the evolution of the genotype-phenotype map itself. That is, the genotypic code and biological development themselves are encoded within organisms, and mutations that alter the structure of the genetic space or genotype-phenotype map may also lead to more or less phenotypic possibilities. In this way, the emergence of a complex evolvable genotypic code and biological development may have been bootstrapped from far simpler reproductive processes by similar non-adaptive mechanisms. In other words, there may be no selective benefit for development or a complex genetic system, which may do no more than potentiate greater phenotypic possibilities. In this way the story of biological evolution may be more fundamentally about an accelerating drive towards diversity than competition over limited resources.

## Methods

The following sections provide more details on the experimental models used in the experiments in this paper. Note that all error bars in figures indicate 95% confidence intervals around the mean, and statistically significant differences are measured by Student's t-test with a p-value of 0.01 unless otherwise noted.

### Abstract Model Details

For both abstract models, at the beginning of the simulation each individual's evolvability was initialized to 0.05. At the beginning of each generation, an individual's niche is perturbed with a probability equivalent to its evolvability, and its evolvability itself is perturbed with a fixed probability (0.01). Changes in evolvability are drawn from a uniform distribution between −0.005 and 0.005.

In the abstract passive drift model, the population consisted of 40,000 individuals that evolved solely due to genetic drift for 3,000 generations. In the model with limited capacity niches, niches were limited to 5 individuals each, and the population was initialized in the first generation with a single individual. Each individual has two offspring in the next generation, which results in geometric population growth except when the niche of an offspring is already filled. Evolution proceeds for 3,000 generations.

### Evolved Robot Model Details

**Figure 10:**
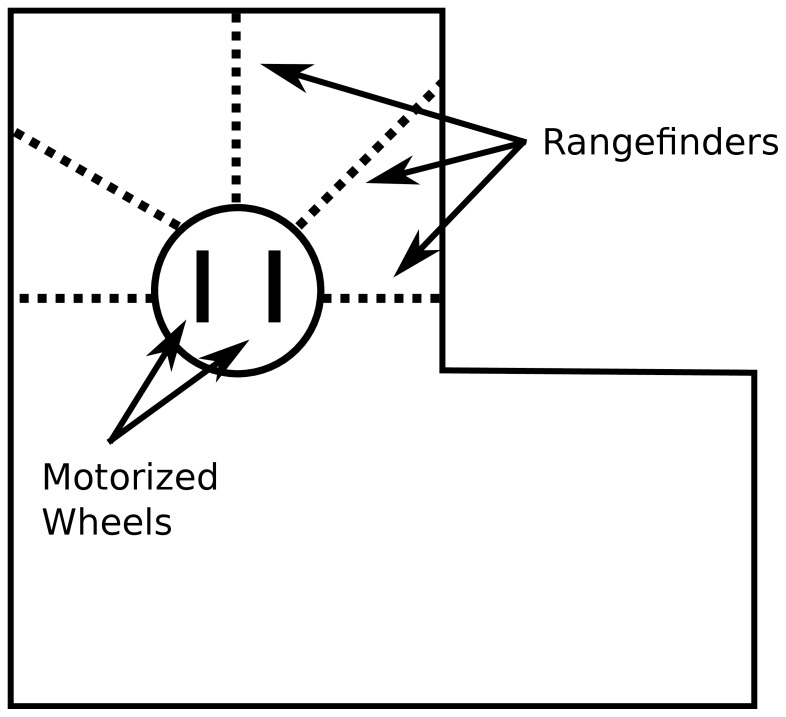
Wheeled robot. The simulated mobile robot is shown that is used in the experiments with evolved machines. Rangefinder sensors allow the robot to perceive obstacles, and the motors controlling its wheels enable the robot to traverse its environment.

**Figure 11:**
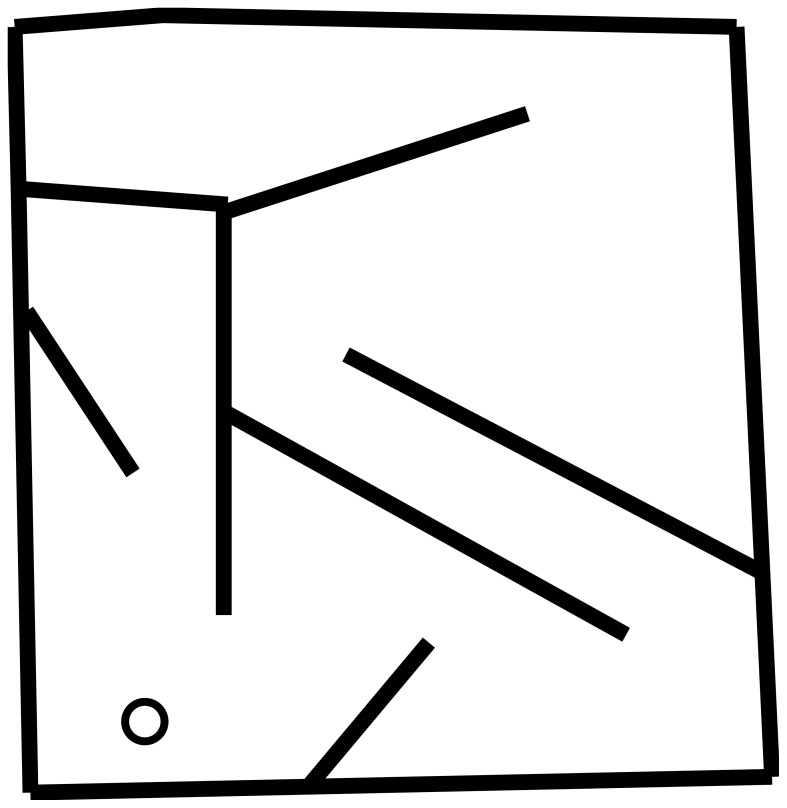
Maze environment. A top-down view of the maze is shown that robots navigate in the experiments with evolved machines. The circle indicates where a robot begins its trial in the maze, which lasts for 400 simulated timesteps.

In all experiments with evolved machines, a genome that maps to an ANN controls a simulated wheeled robot (figure 10) with rangefinder sensors in a maze environment (figure 11). The experimental setup follows prior precedent [14].

A uniform 20×20 grid is superimposed over the maze for calculating robots' niches. A robot's behavioral niche (applied for measuring evolvability in both experiments, as well as to limit population growth in the limited niche capacity experiment) is determined by the grid square within which the robot ends at the termination of an evaluation.

**Figure 12:**
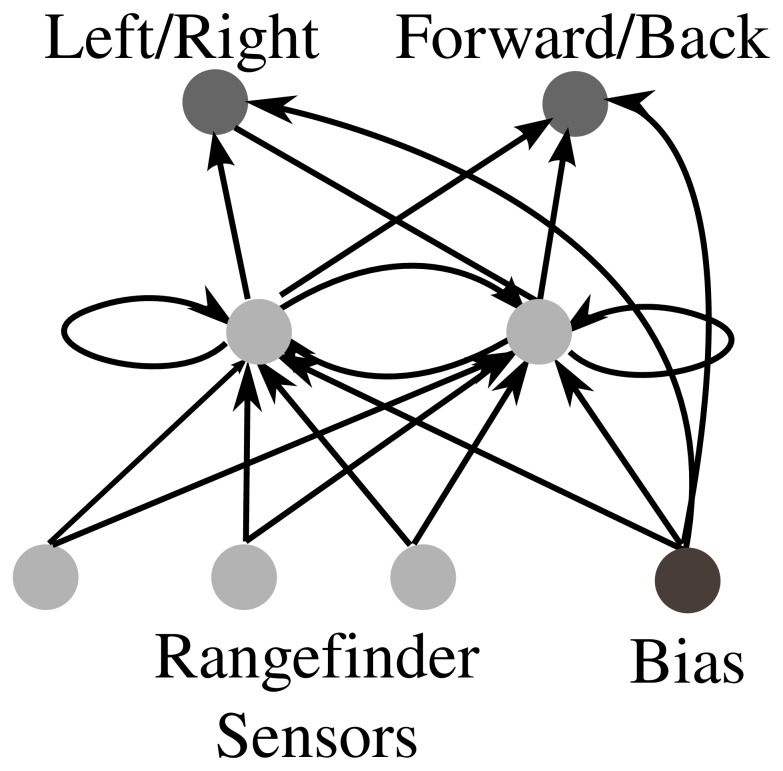
Fixed-topology ANN. The figure illustrates the fully-connected recurrent ANN with 18 possible connections that serves as a space of possible controllers for robots embedded in a maze navigation environment. Each connection can either be excitatory (a weight of 1.0), inhibitory (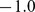) or neutral (0.0). The activation function in the ANN is a steepened sigmoid function [18]. The ANN has three rangefinder sensor inputs, two hidden neurons, and two motor outputs.

The fixed-topology ANN providing the basis for the enumerated genotypic space of ANNs is shown in figure 12. In particular, the genetic space spans variants of a fully-connected recurrent ANN with two input nodes, three hidden nodes, and two output nodes. In total, this kind of ANN has eighteen possible ANN connections that can either be disabled, excitatory, or inhibatory. Thus the space investigated with this model consists of 3^18^, or 38.7 million possible genotypes. The evolvability of each genome is calculated by first enumerating the genomes reachable from it by all possible single connection mutations, and then counting the unique number of behavioral niches that those genomes encode when evaluated in the maze navigation environment.

The drifting model in the enumerated ANN space starts with two million genotypes initialized in each run to the same random starting genotype. The system then drifts for 250 generations.

### Practical ER Model Details

**Figure 13:**
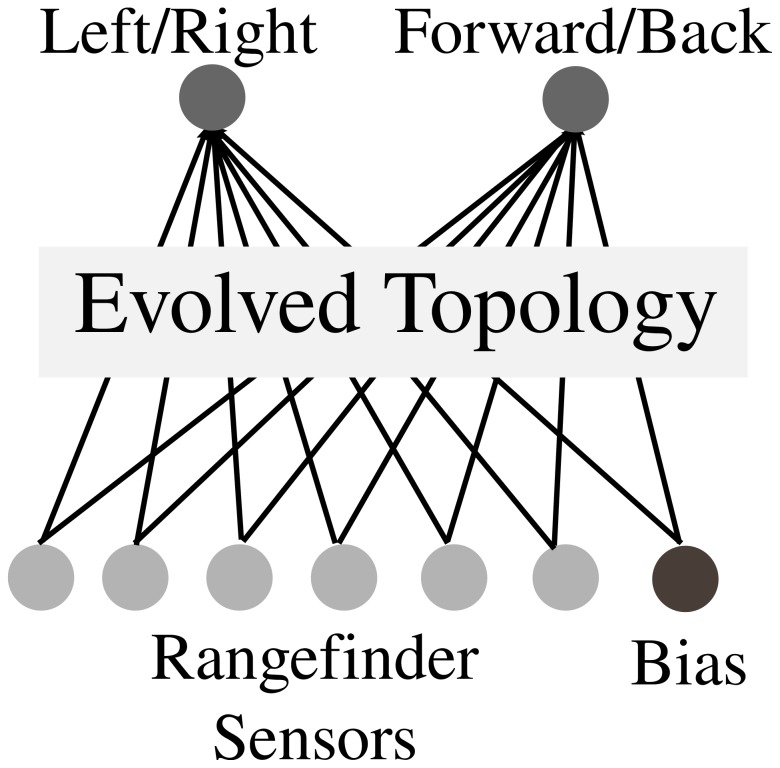
NEAT ANN. The initial topology of the ANN in the practical ER model is shown. Topologies change during evolution from structural mutations that add new nodes and connections. In addition, unlike in the restricted ANN space, connection weights can vary continuously, i.e. weight mutations perturb connections with values drawn from the uniform distribution, and weights are capped between 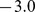 and 3.0.

The practical ER model experiment uses the NEAT algorithm [18], which relaxes the constraints of fixed ANN topology and discrete connection weights. The ANN also provides a greater resolution of sensors, i.e. six rangefinder sensors instead of three. Note that the resolution was reduced in the fixed topology ANN models for combinatorial reasons. The initial NEAT network topology is shown in figure 13. All NEAT parameters are the same as those in Lehman and Stanley [14], which has the same experimental ER maze setup.

Note that the evolvability of a genome is calculated similarly to how it is with the enumerated ANN space, i.e. by counting the unique number of niches encoded by genomes in its mutational neighborhood. However, because *all* reachable mutations cannot be feasibly enumerated in the practical model, instead 200 mutations of a given genome are randomly sampled to generate a reasonable *estimate* of its evolvability.

## References

[1] Dawkins R (2003) The evolution of evolvability. On Growth, Form and Computers: 239–255.

[2] Earl D, Deem M (2004) Evolvability is a selectable trait. Proceedings of the National Academy of Sciences of the United States of America 101: 11531–11536. 15289608 10.1073/pnas.0404656101 PMC511006

[3] Kirschner M, Gerhart J (1998) Evolvability. Proceedings of the National Academy of Sciences of the United States of America 95: 8420. 9671692 10.1073/pnas.95.15.8420 PMC33871

[4] Wagner G, Altenberg L (1996) Complex adaptations and the evolution of evolvability. Evolution 50: 967–976. 28565291 10.1111/j.1558-5646.1996.tb02339.x

[5] Pigliucci M (2008) Is evolvability evolvable? Nature Reviews Genetics 9: 75–82. 10.1038/nrg2278 18059367

[6] Brookfield J (2001) Evolution: The evolvability enigma. Current Biology 11: R106–R108. 11231170 10.1016/s0960-9822(01)00041-0

[7] Wagner A (2008) Robustness and evolvability: a paradox resolved. Proceedings of the Royal Society B: Biological Sciences 275: 91–100. 17971325 10.1098/rspb.2007.1137 PMC2562401

[8] Bloom J, Labthavikul S, Otey C, Arnold F (2006) Protein stability promotes evolvability. Proceedings of the National Academy of Sciences 103: 5869–5874. 10.1073/pnas.0510098103 PMC1458665 16581913

[9] Ancel LW, Fontana W (2000) Plasticity, evolvability, and modularity in rna. Journal of Experimental Zoology 288: 242–283. 11069142 10.1002/1097-010x(20001015)288:3<242::aid-jez5>3.0.co;2-o

[10] Crombach A, Hogeweg P (2008) Evolution of evolvability in gene regulatory networks. PLoS computational biology 4: e1000112. 18617989 10.1371/journal.pcbi.1000112 PMC2432032

[11] Dichtel-Danjoy M, Félix M (2004) Phenotypic neighborhood and micro-evolvability. Trends in Genetics 20: 268–276. 10.1016/j.tig.2004.03.010 15109782

[12] Lenski R, Ofria C, Pennock R, Adami C (2003) The evolutionary origin of complex. Nature. 10.1038/nature01568 12736677

[13] Nolfi S, Floreano D (2000) Evolutionary Robotics. Cambridge: MIT Press.

[14] Lehman J, Stanley KO (2011) Abandoning objectives: Evolution through the search for novelty alone. Evol Comp 19: 189–223. 10.1162/EVCO_a_00025 20868264

[15] Mouret JB, Doncieux S (2012) Encouraging behavioral diversity in evolutionary robotics: an empirical study. Evolutionary Computation 20: 91–133. 21838553 10.1162/EVCO_a_00048

[16] Risi S, Hughes C, Stanley K (2010) Evolving plastic neural networks with novelty search. Adaptive Behavior.

[17] Lehman J, Stanley KO (2011) Improving evolvability through novelty search and self-adaptation. In: Evolutionary Computation (CEC), 2011 IEEE Congress on. IEEE, 2693–2700.

[18] Stanley KO, Miikkulainen R (2002) Evolving neural networks through augmenting topologies. Evolutionary Computation 10: 99–127. 12180173 10.1162/106365602320169811

