# Correction: Evolvability Is Inevitable: Increasing Evolvability without the Pressure to Adapt

**DOI:** 10.1371/annotation/f4c5a0f3-cb53-4c05-a84c-f0aead483b77

**Published:** 2013-05-06

**Authors:** Joel Lehman, Kenneth O. Stanley

There was an error in the Funding statement. The correct statement is:

This research was supported by Defense Advanced Research Projects Agency (DARPA) and Army Research Office through DARPA grant N11AP20003 (Computer Science Study Group Phase 3), and United States Army Research Office grant Award No. W911NF-11-1-0489. This paper does not necessarily reflect the position or policy of the government, and no official endorsement should be inferred. 

